# A 10-Year-Old with Frequent, Disruptive, and Unexplained Night Awakenings

**DOI:** 10.7759/cureus.11893

**Published:** 2020-12-04

**Authors:** Sameh S Morkous

**Affiliations:** 1 Pediatric Neurology, Lehigh Valley Reilly Children's Hospital, Allentown, USA; 2 Pediatrics, Philadelphia College of Osteopathic Medicine & DeSales University, Allentown, USA; 3 Pediatrics, University of South Florida Morsani College of Medicine, Tampa, USA

**Keywords:** child neurology, sleep medicine, epilepsy medicine

## Abstract

A 10-year-old female presented to the sleep clinic for a second opinion about her epilepsy diagnosis. She had been treated with antiepileptic medication but her events persisted. The child would wake up several times every night speaking nonsense words, appear confused to her family, and then go back to sleep. A video of the polysomnography (PSG) showed the patient having two of her typical events. The patient was eventually diagnosed with confusional arousal (CoA) secondary to obstructive sleep apnea (OSA). The nocturnal events resolved after her OSA was treated. This case highlights an atypical clinical presentation for a type of parasomnia like CoA that was misdiagnosed and treated for seizures. It will illustrate OSA and its mechanisms as a potential occasional treatable cause for CoA. It also demonstrates the importance of video-PSG in the work-up of CoA.

## Introduction

The differential diagnosis between sleep-related hypermotor epilepsy (SHE) and parasomnias may be challenging-even for experts in epilepsy and sleep medicine-due to the potential similarities between these two sleep-related manifestations. Misdiagnosis is frequent in SHE patients because of the absence of typical convulsive seizures and the presence of behavioral patterns similar to those observed in non-rapid eye movement (NREM) parasomnias and rapid eye movement (REM) behavior disorders. Background activity on the electroencephalogram (EEG) can be normal in about half of SHE cases creating additional challenges for the appropriate diagnosis. Interictal EEG is normal in about half of cases or may demonstrate rare epileptiform abnormalities enhanced by sleep deprivation and occurring mainly during sleep. The ictal scalp EEG may be normal or may only demonstrate movement artifacts. Epileptiform abnormalities, rhythmic slow activity, or diffuse background flattening over frontal areas are seen in 50-60% of cases. Moreover, the response to antiepileptic drugs is not discriminative for the diagnosis of SHE. A large cohort study reported a diagnostic delay of 12.8±10.1 years in 53.7% of SHE cases with parasomnias being the most frequent misdiagnosis (55.5%). This problematic diagnosis is an important factor that may have an impact over the clinical course of the disease because of potentially inappropriate treatment and management as demonstrated in our case [1].

## Case presentation

A 10-year-old female presented to the sleep clinic for evaluation in regards to her ongoing spells despite being on multiple seizure medications. She had been diagnosed with underlying epilepsy two years ago and was treated with antiepileptic medications, including levetiracetam and oxcarbazepine based on the presentation and the frequent events reported, but her events continued. The episodes, which began four years ago, were now happening twice a night. The child was waking up sporadically from sleep during the night and looked disoriented to the family. During this time the mother reported that the child would utter few unintelligible words rapidly and this was followed by going back to sleep spontaneously. The family reported no motor activity. Epworth Sleepiness Scale (ESS) assessment was 6/24. She did snore with occasional reported respiratory pauses. She had a consistently regular sleep wake cycle with 10-11 hours of sleep and a 9:30 PM bed time. She usually fell asleep within 15 minutes and woke up between 7:30 - 8:00 AM. She had no daily naps. The patient had no other significant medical history. Family history included maternal sleep apnea but no seizures.

Examination revealed patient weight at 37.4kg; height, 139.7cm. Z-scores of 0.63 and 0.27 were based on CDC two to 20 years’ weight-for-age, and stature-for-age data, respectively. Body mass index was 19.15 kg/ (m^2) (Z score: 0.82, 79th percentile). She had a mallampati score of 2; tonsil size of 2+. The remainder of the physical examination was normal.

**Figure 1:**
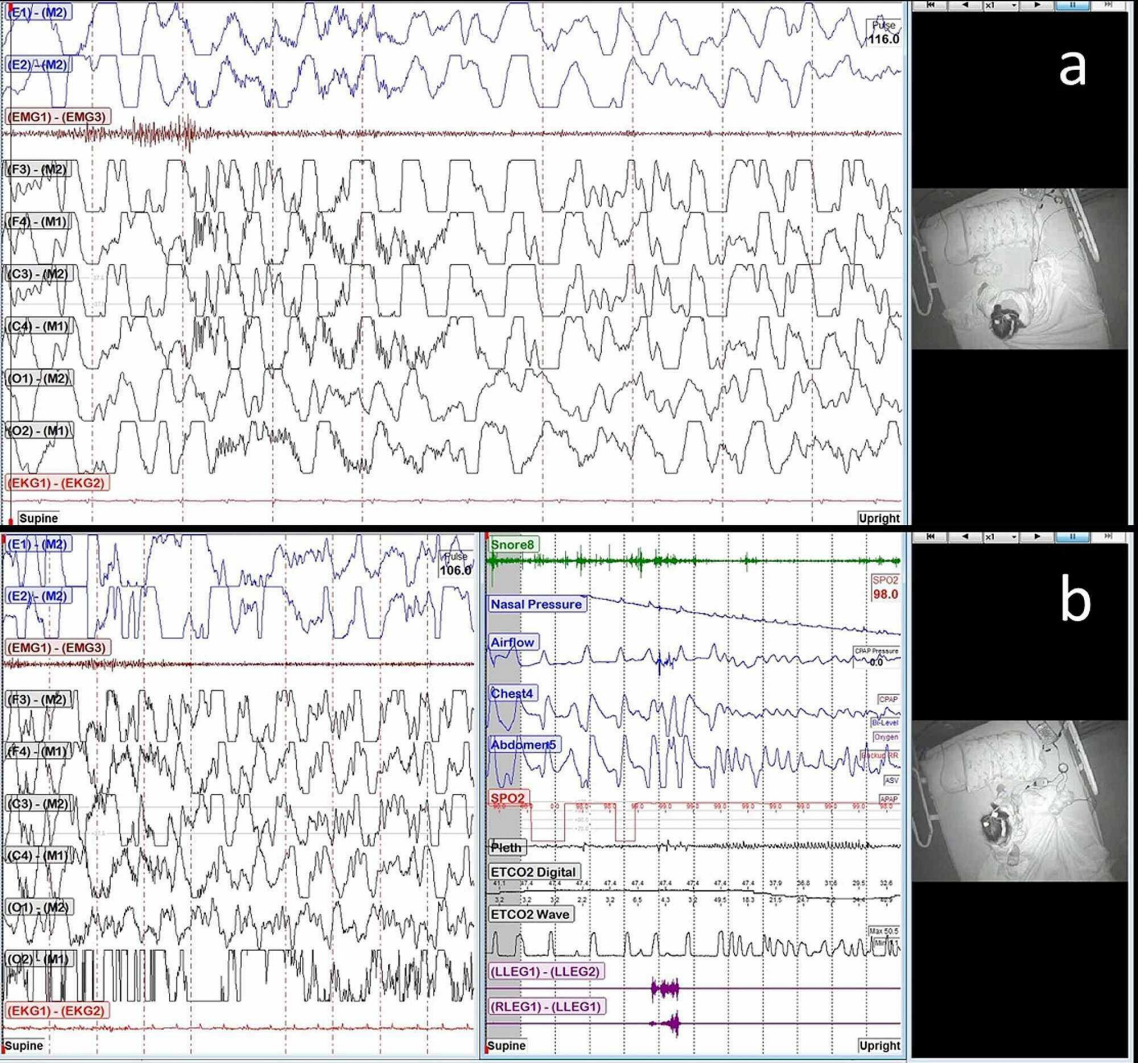
An initial exhibit showing the patient's two typical events (Figure 1A) Event 1: 10-second EEG with patient upright; (Figure 1B) Event 2: 10-second EEG (right) with patient upright and 120- second respiratory channels (left). Sleep architecture was 14.5 minutes (3.4%) in N1, 253 minutes (59.1%) in N2, 108.5 minutes (25.4) in N3, and 52 minutes (12.1%) in R

A nocturnal PSG revealed total sleep time as 428 minutes; lights-off time, 10 PM; lights-on time, 6 AM. The obstructive apnea-hypopnea index was 9 events/h; central apnea index, 0.4 events/h. Sleep latency was 20 minutes, lowest oxygen saturation was 90%, and mean EtCO2 was 35 mmHg. Periodic limb movement index was 0.0 events/h. During the PSG (Figure 1) the patient had 2 typical events at 12:42 AM and 5:24 AM 

Differential diagnosis

The differential diagnosis of paroxysmal events during sleep includes NREM arousal disorders, REM sleep behavior disorder (RBD), psychogenic non-epileptic seizures, sleep-related hypermotor epilepsy (SHE) previously called nocturnal frontal lobe epilepsy (NFLE)-and also temporal lobe seizures. The NREM group includes CoA, sleepwalking, and sleep terrors. General criteria from the International Classification of Sleep Disorders, 3rd Edition, for arousal disorders include (a) recurrent episodes of incomplete awakening, (b) absent or inappropriate responsiveness, (c) limited or no cognition/ dream report, and (d) partial or complete amnesia for the episode. Episodes typically occur several times per month rather than daily as witnessed in this case, which is considered more typical for SHE. Sleep terrors are usually distinguished by diaphoresis and the subsequent lack of recall. Sleepwalking meets the general criteria for NREM arousal disorders and is associated with ambulation [2]. RBD is associated with complex motor behaviors and polysomnographic findings of REM sleep without atonia. The final diagnosis in our case is CoA secondary to moderate OSA. The diagnosis was established by history and the video-polysomnography (vPSG) in our patient.

Treatment

Confusional arousal is typically managed by avoiding triggers like sleep deprivation, treating the underlying sleep disorders (OSA or restless legs syndrome), behavioral modifications, and rarely, pharmacological treatment. There are no evidence-based treatment guidelines at present; however, low doses of a benzodiazepine or selective serotonin reuptake inhibitors have been used [3] -especially if there is a concern about injury to self or others-or they can be considered if the CoA persists.

Outcome and follow up

A follow-up ambulatory 48-hour EEG and brain MRI was originally ordered by the referring provider for the initial concerns regarding her seizures. Both were reported normal by the ordering provider but no events were captured at the time. This also highlights the critical role of continuous video EEG monitoring (cVEEG) to make the diagnosis-as reported by Pavlakis and Douglass [4] . Tonsillectomy and adenoidectomy (T&A) was performed. On 3-month follow-up, her events had resolved with no concerns for snoring or witnessed apnea reported by the family. Thus, no additional PSG following the T&A was done. Family counseling, environmental safety, the importance of addressing any possible triggers, and sufficient sleep for age were discussed with the family.

## Discussion

Few cases in the literature have reported the possible association between parasomnia and OSA, and those primarily involved sexsomnia and parasomnia overlap disorder (POD). The first case described an adolescent boy who developed sexsomnia [5]. The patient had four episodes of stereotypical masturbatory behaviors during the initial sleep study each occurring after arousals triggered by obstructive apnea events out of REM sleep. In this case, there have been modest reductions in the frequency of sleep-related sexual behaviors on CPAP. Another case report is a 42-year-old with parasomnia overlap disorder (POD) who had two notable parasomnia findings during vPSG with arousal out of stage N3 sleep [6]. During this, the patient moved his legs violently and the muscle tone increased overall through stage REM sleep with several episodes of yelling profanities and kicking.

Neither of these case reports demonstrates the association between CoA and OSA on the vPSG. Despite this possible overall association between parasomnia and OSA [7], few published cases have illustrated the association or mechanisms between CoA and OSA like ours does. This case also demonstrates that CoA can occur at any time of night rather than the typically reported early night time. It can also occur frequently every night. Both of these confounding variables contributed to the misdiagnosis of underlying epilepsy rather than a parasomnia in the context of a potentially treatable trigger like OSA as exhibited in this patient.

**Figure 2:**
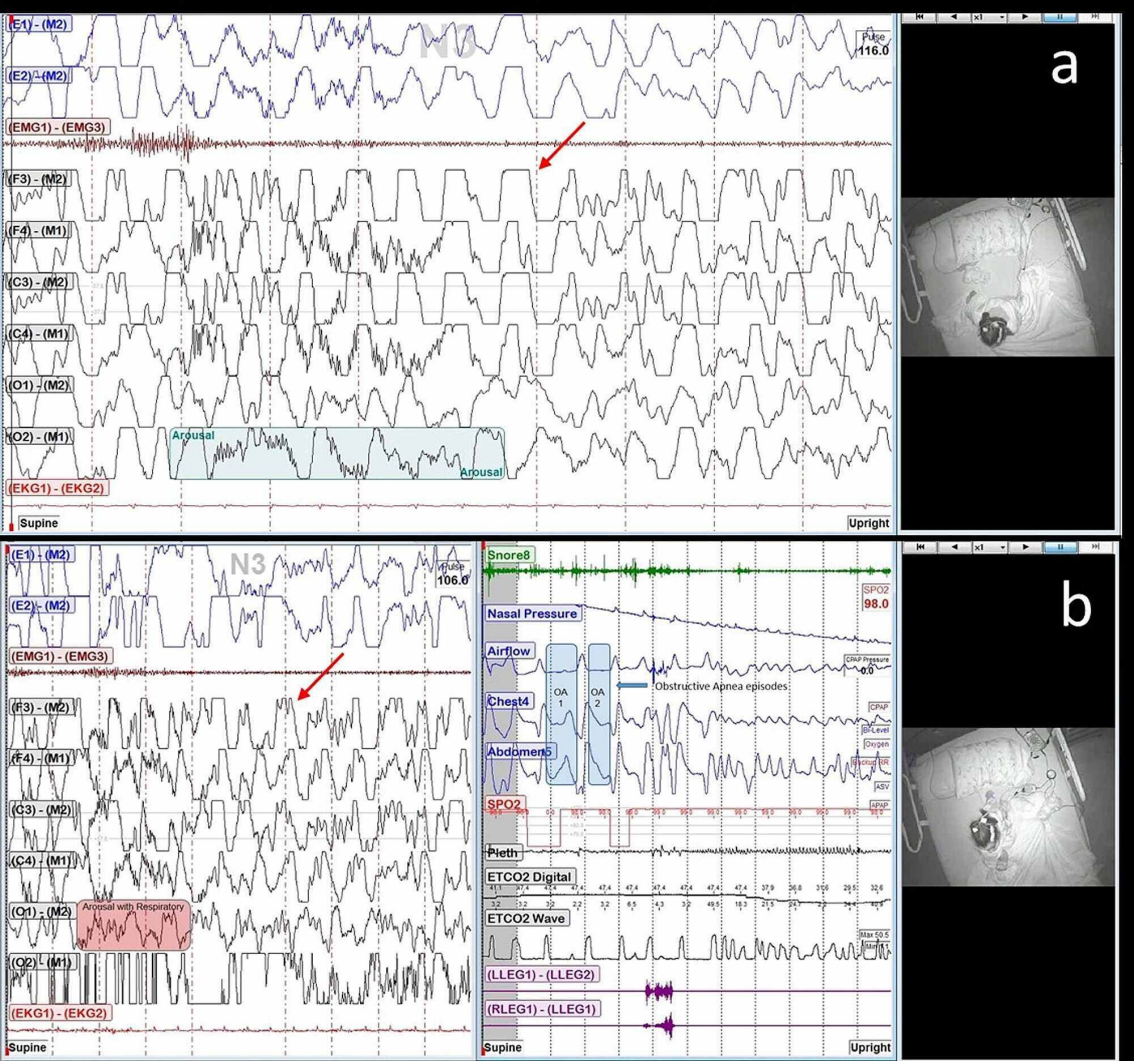
The events occurring during stage N3 with the associated mechanisms demonstrated (Figure 2A)  Event 1: 10-second EEG with arousal event highlighted; (Figure 2B)  Event 2: 10-second EEG (right) with patient upright and with the arousal highlighted, and 120-second respiratory channels (left) with the obstructive sleep apnea (OSA) respiratory events highlighted. Red arrows indicate examples of persistent delta activity.

Differentiating between nocturnal seizures and NREM parasomnias can be challenging. Sleep-related hypermotor epilepsy is focal epilepsy with the seizures characterized by asymmetric tonic/dystonic posturing and/or complex hyperkinetic seizures occurring mostly during sleep [1] and usually occur out of stage N2 [8]. However, in our patient, both of the events occurred during stage N3 with no hypermotor activity and were characterized by confusion for about one minute following the arousal (Figure 2). The semiology and the EEG findings are consistent with CoA.

**Figure 3:**
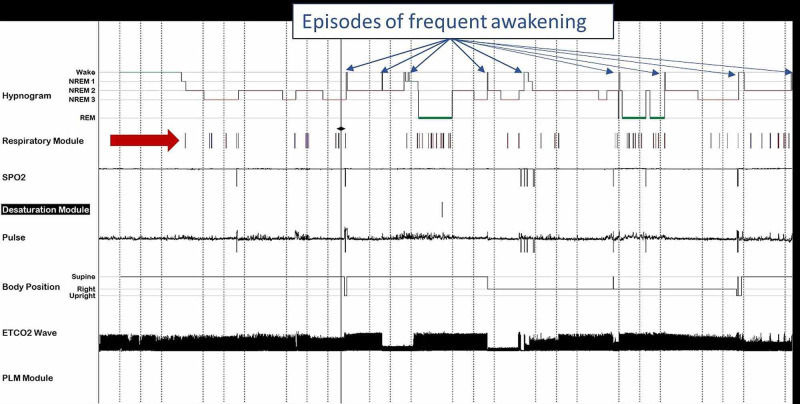
The hypnogram showing evidence of the Obstructive Sleep Apnea (OSA) Hypnogram of the patient with the respiratory events noted by the red arrow and episodes of frequent awakening indicated by blue arrows. The Polysomnography (PSG) shows evidence of sleep fragmentation: sleep efficiency was 83%, arousal index was 16.5/h, and with 22 awakening

The PSG showed evidence of moderate OSA (Figure 3). Parasomnia has been linked to OSA [9], most likely related to arousals triggered by respiratory events. Lundetrae reported that respiratory events tend to occur more frequently in children with parasomnia than in controls and with an arousal reaction probably involved in triggering the parasomnia [7]. OSA is known to cause sleep fragmentation, which leads to the expression of parasomnia [9]. Both of these described mechanisms were observed in our patient. An arousal alone was triggered spontaneously in event one (Figure 2A), while an arousal followed by an OSA event occurred in event two (Figure 2B). Both episodes were associated with the patient sitting upright.

Confusional arousal is common in children below age 13 with a prevalence of 17.3% [3]. It is characterized by disoriented behavior during an arousal from NREM sleep often with poor recall of events. Confusional arousal lasts between five and 15 minutes and is typically benign although the occasional patient can become aggressive and violent [10]. Confusional arousal usually occurs from stage N3 in the first part of the night but can also occur after an arousal from NREM sleep at any hour as exhibited in this case. The high-amplitude slowing during CoA events is considered a hallmark of arousal disorders although it is non-specific [11]. They can also occur out of stage N2, especially in adults [7]. Disorders of arousal are common phenomena in childhood, tend to decrease with age [12], and become less frequent after the age of five [13]. However, NREM parasomnias can also persist into adulthood and in some cases can even start de novo in adulthood. The reasons for the variable onset and continuity of NREM parasomnias are unknown [14], thus both adult and pediatric providers need to be aware of this phenomena.

## Conclusions

This case highlights a peculiar clinical presentation for episodic disturbances related to sleep (parasomnias) like CoA where she will have frequent nocturnal events that will occur sporadically throughout the night rather than the more typical presentation for CoA with the spells usually occurring in the first part of the night and only several times per month. We, then, display the important role of the video-PSG in the work-up of parasomnias and how this can be utilized to differentiate an underlying parasomnia from a seizure disorder. Recognizing all of this in patients by providers is pivotal to differentiate parasomnias from underlying seizures and thus avoid any possible erroneous diagnosis and/or subsequent unnecessary use of seizure medications similar to the clinical context that was discussed.

Confusional arousal is generally managed by educating the families and implementing environmental safety measures as mentioned earlier. However, it is also important to identify and treat the predisposing factors including other sleep disorders like OSA that can trigger CoA. This case reinforces this relation and demonstrates the underlying mechanisms by which OSA trigger CoA. The patient improved after the OSA was treated. This emphasizes the importance of recognizing, evaluating for, and subsequently treating OSA by providers as a potentially treatable culprit to improve an underlying parasomnia like CoA.

## References

[1] Bisulli F, Licchetta L, Tinuper Tinuper, P P (2019). Sleep related hyper motor epilepsy (SHE): a unique syndrome with heterogeneous genetic etiologies. Sleep Sci Pract.

[2] Sateia MJ (2014). International classification of sleep disorders-third edition: highlights and modifications. Chest.

[3] Berry RB (2012). Fundamentals of sleep medicine. https://www.elsevier.com/books/fundamentals-of-sleep-medicine/berry/978-1-4377-0326-9.

[4] Pavlakis PP, Douglass Douglass, LM LM (2015). Pearls & Oy-sters: a case of refractory nocturnal seizures. Neurology.

[5] Contreras JB, Richardson J, Kotagal S (2019). Sexsomnia in an adolescent. J Clin Sleep Med.

[6] Soca R, Keenan JC, Schenck CH (2018). Parasomnia overlap disorder with sexual behaviors during sleep in a patient with obstructive sleep apnea. J Clin Sleep Med.

[7] Lundetræ RS, Saxvig IW, Pallesen S, Aurlien H, Lehmann S and Bjorvatn B (2018). Prevalence of parasomnias in patients with obstructive sleep apnea. A registry-based cross-sectional study. Front Psychol.

[8] Tinuper P, Bisulli F, Cross JH, Hesdorffer D, Kahane P, Nobili L. Berkovic S, Ottman R (2016). Definition and diagnostic criteria of sleep-related hypermotor epilepsy. Neurology.

[9] Chen YH, Keller JK, Kang JH, Hsieh HJ, Lin HC (2013). Obstructive sleep apnea and the subsequent risk of depressive disorder: a population-based follow-up study. J Clin Sleep Med.

[10] Howell MJ (2012). Parasomnias: an updated review. Neurotherapeutics.

[11] Perez H, Santamaria J (2017). Sudden awakening during polysomnography. Ann Am Thorac Soc. Sleep Fragments [cited.

[12] Loddo G, Lopez R, Cilea R (2019). Disorders of arousal in adults: new diagnostic tools for clinical practice. Sleep Science Practice 3.

[13] Markov D, Jaffe F, Doghramji K (2006 Jul). Update on parasomnias: a review for psychiatric practice. Psychiatry (Edgmont.

[14] Hrozanova M, Morrison I, Riha RL (2019). Adult NREM Parasomnias: an update. Clocks & Sleep.

